# Long-term follow-up of modified shunt-restricted instep arterialized venous flap for reconstruction of hand defects

**DOI:** 10.3389/fmed.2025.1662159

**Published:** 2025-08-18

**Authors:** Wei Chen, Yang Jian, Maolin Cheng, Runxue Guan, Wenyu An, Chenglan Yang, Jian Zhou, Shujun Li, Shusen Chang, Chengliang Deng, Kaiyu Nie, Zairong Wei

**Affiliations:** ^1^Department of Burns and Plastic Surgery, Affiliated Hospital of Zunyi Medical University, Zunyi, China; ^2^The 2011 Collaborative Innovation Center of Tissue Damage Repair and Regeneration Medicine, Affiliated Hospital of Zunyi Medical University, Zunyi, China; ^3^The Collaborative Innovation Center of Tissue Damage Repair and Regeneration Medicine, Zunyi Medical University, Zunyi, China; ^4^Guizhou Biofabrication Laboratory, Affiliated Hospital of Zunyi Medical University, Zunyi, China

**Keywords:** venous flap, flap, hand defect, arterialized venous flap, reconstruction

## Abstract

**Background:**

Instep arterialized venous flaps (iAVF) are suitable for reconstructing soft tissue defects of the hand while concurrently fulfilling aesthetic requirements. However, iAVF still face challenges such as unstable survival rates and swelling. Thus, this study aimed to propose a new strategy for iAVF, namely a modified shunt-restricted iAVF, for the reconstruction of hand defects.

**Methods:**

This retrospective study included 16 patients who underwent reconstruction of hand defects using the modified iAVF approach. All flaps were designed with antegrade flow, and the direct communicating branches between the afferent and efferent veins of the flap were ligated by sutures. The donor site of the flap was repaired by tension-reduction suture or full-thickness skin graft. Patient satisfaction and the Michigan Hand Questionnaire (MHQ) were used to evaluate the reconstructive effect and feasibility of the technique.

**Results:**

The thickness of the flaps ranged between 1.6–3.0 mm, with an average thickness of 2.3 mm. The donor site was directly sutured in 2 cases and repaired by full-thickness skin graft in 14 cases. All flaps completely survived. The flap color was pale in 11 cases and transitioned to a ruddy color within 2–5 h post-operatively, with an average of 3.3 h. Moreover, 12 cases developed mild swelling within 2 weeks postoperatively, whilst 4 cases presented with sporadic blisters. The follow-up duration ranged between 2 and 4.83 years (mean 3.71 years). The color and texture of the flap were close to healthy skin, and joint function was satisfactorily recovered. The mean total MHQ score for the injured side was similar to that for the contralateral healthy side (99.40 ± 1.72 vs. 99.96 ± 0.15, *p* = 0.068; 95% confidence interval: 0.0–0.38). No significant differences were noted in MHQ scales.

**Conclusion:**

Modified iAVF represents an aesthetic and functional superthin flap, which is simple and reliable for hand defect reconstruction.

## 1 Introduction

Arterial flaps, including pedicled island flaps and free flaps, are the most commonly used for hand reconstruction. Recently, Delle Femmine and her colleagues introduced an adipofascial flap for distal finger and nail-bed defects (1, 2). Nevertheless, all of these flaps require arterial sacrifice and may result in post-operative bulkiness (3–5). Venous flaps (VFs) are considered the optimal source for the reconstruction of soft tissue defects in hands and feet owing to its thin texture and flexibility (6–8), low risk of complications at the donor site, ease of harvesting, possibility of harvesting chimeric flap (incorporating tendons (9, 10) and nerves (8, 11)) and preservation of primary blood vessels at the donor site (6, 8, 10, 12). However, VFs are generally not the first choice for microsurgical reconstruction due to their poor survival rates and associated risk of congestion, swelling, partial necrosis, and difficulties associated with postoperative monitoring.

Several strategies have been used to improve the survival rate of VF and minimize complications (13). Among them, arterialized venous flaps (AVF) are frequently employed, wherein arterial blood flows into afferent veins and drains through efferent veins into recipient veins (12). AVF can be categorized into antegrade and retrograde AVF (13). The direction of arterial inflow in antegrade AVF is consistent with that of the venous valve, allowing unimpeded blood flow; nonetheless, this can result in hypoperfusion and subsequently flap necrosis (8). In contrast, the arterial inflow direction in the retrograde AVF opposes that of the venous valve, consequently increasing blood perfusion into the edge of the flap and the viable area of the flap (6, 14). However, studies have reported no significant difference in the survival rate between retrograde and anterograde AVFs (4). Moreover, retrograde AVF typically requires pre-treatment of venous valves; otherwise, intact venous valves may obstruct blood flow and increase the risk of flap necrosis (15–17). In addition, three two-stage methods have been developed to improve flap perfusion, including dilated VF, delayed VF, and pre-arterialized VF (13). Despite these methods having improved the survival rate of VF, they have not been widely adopted due to the prolonged time and inconsistent outcomes (13). At present, a restricted shunt is a commonly used strategy to increase perfusion pressure, reduce the risk of venous congestion, and improve the survival rate and quality of the flap (7, 18, 19).

Instep AVF (iAVF) offers substantial advantages for the reconstruction of soft tissue defects in the hands and wrists, such as a skin texture similar to the defect, easy-to-hide scars, the ability to carry tendons and nerves (11, 20–22). However, the dorsum of the foot is one of the least commonly used donor sites (12), with a complete survival rate of 66.7% and a partial necrosis rate of 33.3% (23). To improve the survival rate of iAVF, Yu et al. (21) performed retrograde iAVF to improve the survival rate of iAVF and reduce the risk of complications, and all 6 flaps survived. They identified venous congestion as a prevalent complication. Ling et al. (4) performed iAVF with a U-shaped venous configuration to repair finger defects and noted no difference in survival rate between anterograde and retrograde AVFs. Moreover, retrograde iAVF is linked to delayed-onset swelling and insufficient blood supply (4). Restricted shunt may be a potential method to solve the above problems and improve the survival rate of iAVF.

We have previously reported on modified shunt-restricted forearm AVF for repairing hand electrical injuries, showing the reliability of this approach (24). Consequently, to improve the reliability of iAVF and concomitantly minimize the risk of complications, a modified iAVF protocol was designed for the reconstruction of skin and soft tissue defects of hands.

## 2 Patients and methods

### 2.1 Case series

This study was conducted in accordance with the Declaration of Helsinki, and informed consent was signed by all patients prior to the surgical intervention. The study was approved by the Ethics Committees of Affiliated Hospital of Zunyi Medical University (KLLY-2021-182). This study was conducted according to the Preferred Reporting of Case Series in Surgery (PROCESS) Criteria (25).

From January 2020 to December 2021, 16 patients, comprising 13 males and 3 females, with unilateral hand defects underwent modified iAVF reconstruction at the Affiliated Hospital of Zunyi Medical University. The age range was 25–54 years, with a mean of 44.1 years. The interval between injury and surgery ranged between 0.5–6 h (mean 3.5 h). Two patients had injuries involving two fingers, whereas the remaining patients experienced injuries to a single finger. Specifically, the injured areas included 6 thumbs, 7 index fingers, 1 middle finger, and 4 dorsal hand defects (Table 1). The soft tissue defect area ranged from 2 cm × 2 cm to 10 cm × 6 cm. All wounds involved exposed tendons or bone, and 3 cases included partial defects of the extensor tendon of the finger.

**Table 1:** Clinical characteristics.

No.	Age (years)/Sex	Site of injury	Injure duration (h)	Follow up (years)
1	45/F	Palmar side of left thumb	3.0	3.25
2	36/M	Dorsal side of left thumb and index finger	0.5	4.42
3	53/F	Dorsal side of left index finger	3.0	3.67
4	45/M	Dorsal side of right hand	6.0	2.00
5	31/M	Dorsal side of right index and middle finger	5.5	3.50
6	46/F	Ulnar side of left index finger	1.0	3.50
7	47/M	Dorsal side of right hand	2.0	4.83
8	47/M	Dorsal side of left index finger	3.5	3.92
9	54/M	Palmar side of left thumb	3.0	3.33
10	49/M	Volar and dorsal side of the right thumb	4.0	4.17
11	41/M	Dorsal side of the left hand	5.0	3.83
12	49/M	Dorsal side of the left hand	0.5	4.00
13	52/M	Dorsal side of left index finger	4.0	3.08
14	35/M	Palmar side of right thumb	5.5	3.58
15	25/M	Dorsal side of right index finger	3.0	3.83
16	51/M	Palmar side of left thumb	6.0	4.42

M: male; F: female.

### 2.2 Preoperative evaluation

Emergency debridement was performed for all patients following admission. Additionally, cephalosporin antibiotics were administered to prevent infection, and modified iAVF reconstruction was performed 2–5 days (mean 3d) following admission. Criteria for employed modified iAVF reconstruction were as follows: (1) the wound was not severely contaminated; (2) defects of the hands and wrists that could be reconstructed using local or pedicled flaps; (3) small- and medium-sized hand defects; (4) the patient consented to undergo modified iAVF reconstruction.

### 2.3 Surgical procedures

#### 2.3.1 Flap design and harvesting

The operation was performed under general anesthesia with endotracheal intubation. A rubber tourniquet was applied at the mid-calf level to engorge the dorsalis pedis vein. The modified iAVF was designed according to the dorsalis pedis vein and the recipient site. The distal and proximal ends of the flap were marked, and the axis of the flap was consistent with the dorsalis pedis vein. Next, the rubber tourniquet was removed, following which an air-pressure tourniquet was placed around the upper middle third of the thigh and inflated. Afterward, skin and subcutaneous tissues were incised following the preoperative design. The flap was harvested at the level of the superficial fascia while preserving the cutaneous nerve of the dorsum of the foot. The length of the vascular pedicle was tailored according to the requirements of the recipient site.

#### 2.3.2 Modified restricted shunt

**Figure 1:**
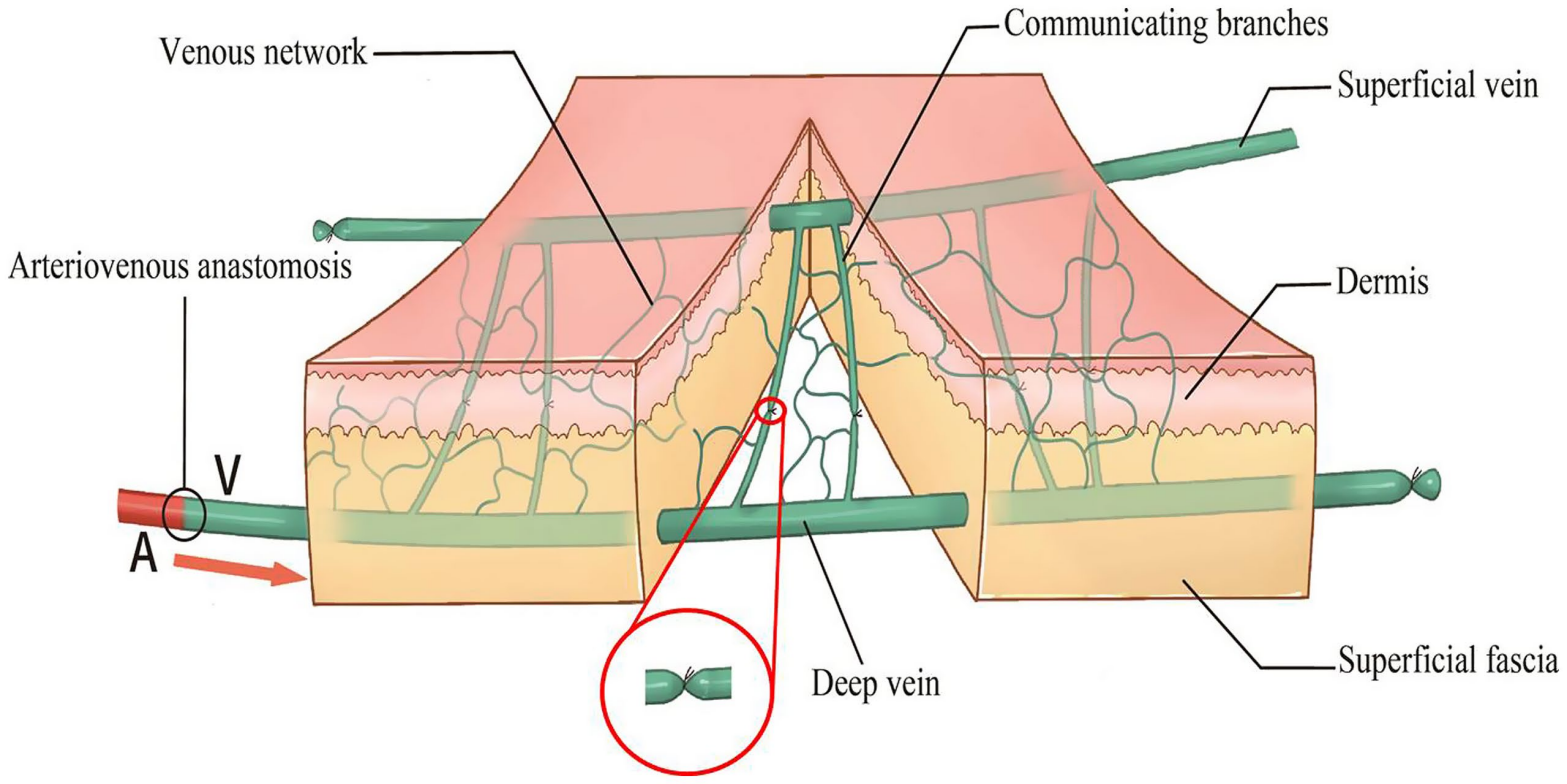
Diagram of an improved restricted shunt technique. The right side is the distal end of the flap, and the left side is the proximal end of the flap.

The following optimizations were incorporated (Figure 1): (1) At the distal end of the flap, a small-diameter deep vein was retained as the afferent vein and ligated at the proximal end. (2) The subdermal vein (superficial) with a larger diameter at the proximal end of the flap served as the efferent vein. Generally, one vein was retained for smaller flaps, whereas over 2 veins were retained for larger flaps to minimize the risk of dermal venous congestion. (3) Under a 4-fold microscope, the communicating branches between the superficial and deep venous systems were ligated to block blood flow. The direct communicating branch between the afferent and efferent veins was ligated in the middle part of the flap to limit arteriovenous shunt and ensure that the arterialized afferent vein could provide sufficient arterial blood for microcirculation and flap survival. (4) The length of the afferent vein was minimized to reduce the number of venous valves in the pedicle of the flap vessel. (5) The length of the efferent veins was individualized to meet the requirements of the vascular pedicle.

#### 2.3.3 Antegrade transplantation

After completing the modified restricted shunt of the flap, AVF was transplanted using an anterograde approach, wherein the distal afferent vein of the flap was end-to-end anastomosed to the recipient artery to ensure that the direction of arterial blood inflow was consistent with that of venous valves. Meanwhile, the proximal efferent vein of the flap was anastomosed with the dorsal digital or dorsal metacarpal veins in the recipient site. The flap was sutured without tension, and a rubber piece was placed under the flap. The donor site was repaired using either tension-reduction sutures or a full-thickness skin graft (FTSG) harvested from the groin, according to the size of the defect.

#### 2.3.4 Postoperative management

Flap color, surface temperature, capillary refill time, and bulla formation were observed and recorded. Antibiotics were administered for 48–72 h, whilst antivasospastic drugs and anticoagulants were given for 5–7 d. The rubber piece was removed 2 d post-operatively, the sutures were removed 2 weeks post-surgery, and functional exercises were initiated 3 weeks after surgery. During follow-up, the reconstructive outcomes and feasibility of the technique were evaluated by assessing patient satisfaction and the Michigan Hand Questionnaire (MHQ), which is a self-assessment tool for evaluating hand function and covers the following domains: overall hand function (OHF), activities of daily living (ADL), pain, work performance (WP), aesthetics, and patient satisfaction with hand function (26). On the pain scale, higher scores reflect greater pain levels. On the other five scales, higher scores indicate better hand performance. Notably, MHQ has been used to evaluate functional outcomes after VF reconstruction of upper limb defects (27).

### 2.4 Statistical analysis

Normally distributed data were expressed as the mean ± standard deviation (SD), whereas non-normally distributed data were presented as the median. The MHQ results were compared using the Wilcoxon Rank-Sum Test with a 95% confidence internal (CI). *p* < 0.05 was considered statistically significant.

## 3 Results

The sixteen patients who underwent modified iAVF achieved complete flap survival (Table 1). The flap areas ranged from 2.5 cm × 2 cm to 10 cm × 6.5 cm, with 2 cases requiring multi-paddled flaps. The thickness of the flap ranged between 1.6–3.0 mm, with an average of 2.3 mm. The donor site was directly closed in 2 cases and repaired using FTSG in 14 cases. At the end of the operation, the color of 5 and 11 flaps was red and pale, respectively, with the flap color displaying a ruddy complexion within 2 to 5 h postoperatively, with an average of 3.3 h (Table 2). During the early postoperative stage, minor swelling was noted, with no signs of tension blisters, and flap color was similar to healthy tissue. Interestingly, swelling exacerbated 72 h postoperatively in 4 cases (Table 2), accompanied by a purplish red discoloration and the presence of sporadic blisters, which improved after dressing change. After 1–2 weeks, the swelling subsided, and the color steadily turned red. FTSG was completely viable, and scar hyperplasia was observed in some patients. Motor function of the foot remained fully intact. Moreover, only a linear scar was left in the groin. Patients were followed up via telephone and outpatient visits for 2–4.83 years, with an average of 3.71 years (Table 1). As anticipated, flap color and texture were comparable to local healthy skin, and functional recovery was satisfactory (Supplementary Table 1). All patients were right-hand dominant, and the MHQ score of the injured hand was 100 (93.22, 100), which was comparable to that of the healthy hand (100) (*p* = 0.068, 95% CI: 0.0–0.38). Likewise, the scores for OHF, ADL, total ADL, pain, WP, aesthetics, and satisfaction were 100 (95.00, 100), 100 (95.00, 100), 100 (93.93, 100), 0 (0, 10), 100 (95.00, 100), 100 (93.75,100), and 100 (91.67, 100) on the injured side and 100 (*p* = 0.317, 95% CI: 0.83–1.0), 100 (*p* = 0.157, 95% CI: 0.54–0.96), 100 (*p* = 0.180, 95% CI: 0.14–0.61), 0 (*p* = 0.180, 95% CI: 0.14–0.61), 100 (*p* = 0.317, 95% CI: 0.83–1.0), 100(*p* = 0.317, 95% CI: 0.83–1.0), and 100(*p* = 0.317, 95% CI: 0.83–1.0) on the healthy side, respectively, and no significant differences were noted across any scale (Supplementary Table 1).

**Table 2:** Details of the surgery.

No.	Tension blisters	Immediate state	Time of flap reddening (h)	Operative time (h)	Closing the donor site	Thickness of flap (mm)	Size of flap (mm)
1	No	Pallid	2	3.5	FTSG	3.0	50 × 18
2	No	Pallid	2.6	4.8	FTSG	2.5	100 × 65
3	No	Ruddy	0	3.8	FTSG	2.0	50 × 25
4	No	Pallid	2.8	2.6	FTSG	2.0	50 × 20
5	Yes	Pallid	3.2	4.8	FTSG	2.6	75 × 50
6	No	Ruddy	0	6.0	FTSG	1.8	50 × 20
7	No	Pallid	4.2	4.7	FTSG	3.0	85 × 80
8	Yes	Pallid	3.6	7.2	FTSG	2.0	40 × 20
9	No	Ruddy	0	3.4	Suture	2.5	25 × 20
10	Yes	Pallid	4	5.6	FTSG	2.5	60 × 50
11	No	Pallid	2.5	3.1	FTSG	1.6	60 × 60
12	No	Ruddy	0	4.6	FTSG	2.0	60 × 45
13	No	Pallid	0	3.7	FTSG	3.0	40 × 13
14	Yes	Pallid	3	4.8	FTSG	1.8	65 × 55
15	No	Ruddy	5	3.0	FTSG	2.6	45 × 25
16	No	Pallid	3	4.1	Suture	2.0	35 × 20

FTSG: full-thickness skin graft.

### 3.1 Typical case

**Figure 2:**
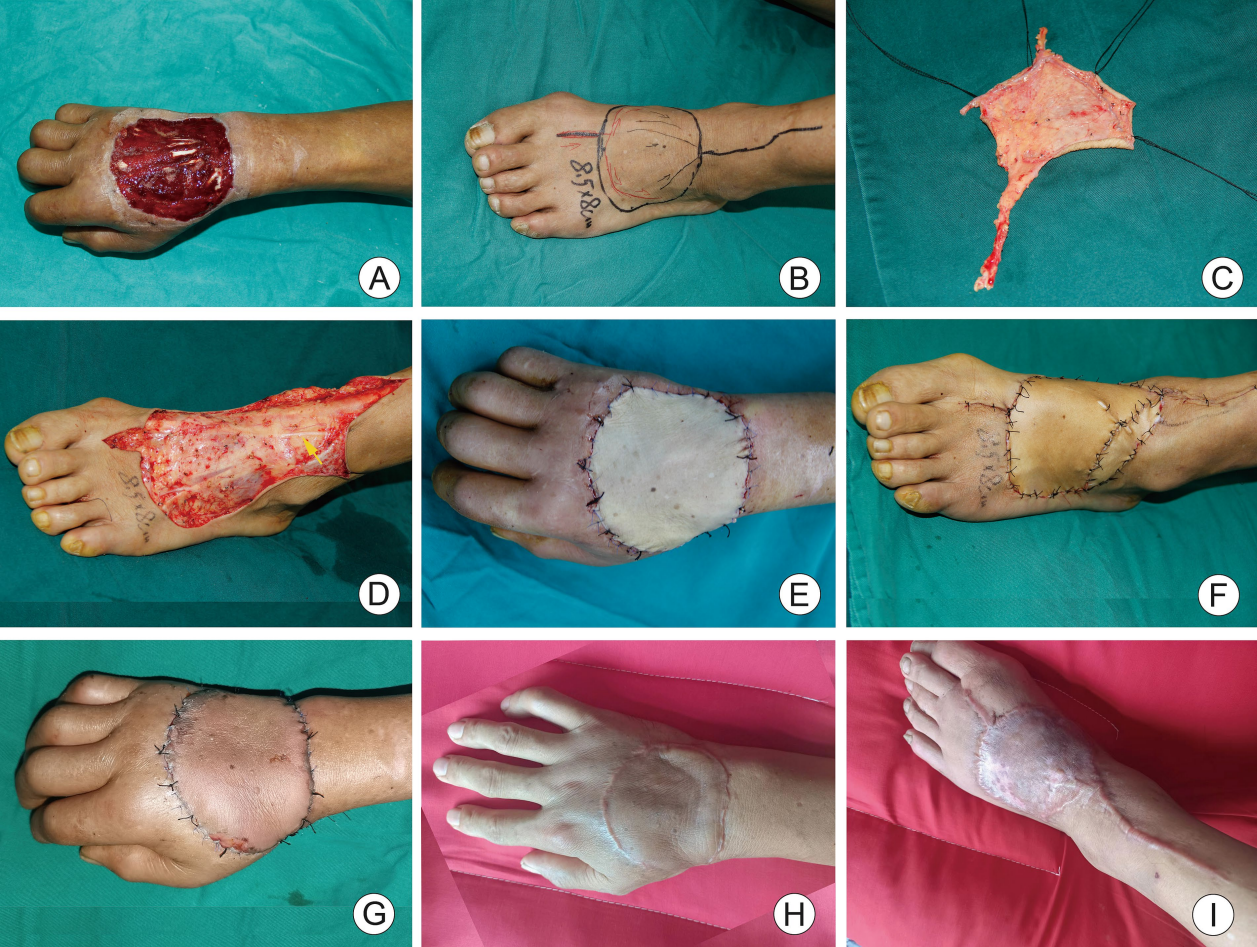
A 47-year-old male with skin defect on the back of the right hand. **(A)** After 5 days of negative pressure wound therapy, skin defect on the back of the right hand and tendon exposure. **(B)** Modified dorsalis arterialized venous flaps was designed for the dorsum of left foot. **(C)** Shunt-restricted operation. **(D)** The dorsal cutaneous nerve of foot was preserved (yellow arrow). **(E)** Immediate postoperative appearance of recipient area. **(F)** Immediate appearance of ipsilateral inguinal full-thickness skin graft at donor site. **(G)** Two weeks after surgery, the flap was ruddy without significant swelling. **(H,I)** Appearance of recipient and donor site 6 months after surgery.

A 47-year-old male patient attended the hospital with a skin defect on the dorsum of the right hand caused by mechanical entanglement injury as the chief complaint. Emergency debridement and negative pressure wound therapy were performed under brachial plexus anesthesia. After 5 days, the negative pressure device was removed, revealing a skin and soft tissue defect area of the right dorsum of 8 cm × 7.5 cm with exposed muscle and tendons (Figure 2). A modified iAVF was designed and harvested on the dorsum of the left foot, measuring 8.5 cm × 8 cm (Figure 2). Microscopic restricted shunt was performed (Figure 2). The AVF was transplanted using the anterograde approach, with an afferent-to-efferent vein ratio of 1:1. Thereafter, the wound in the donor site of the flap was repaired using an FTSG harvested from the ipsilateral groin (Figure 2). Interestingly, the flap appeared ruddy immediately after the surgical intervention (Figure 2). However, marginal swelling was noted during hospitalization, which subsided within 2 weeks without blister formation. At the six-month follow-up, the flap remained viable, with an appearance similar to the surrounding skin (Figure 2).

## 4 Discussion

This case series aimed to outline our innovation and experience with 16 surgical procedures for the reconstruction of hand defects using modified iAVF. Herein, the shunt-restricted method proposed by Lin et al. (18) was modified and applied. Blocking the communicating branches between the superficial and deep veins and the direct communicating branches in the middle part of the flap increased both peripheral perfusion and flap survival rate. In addition, veins with smaller diameters were selected as afferent veins, whereas veins with larger diameters served as efferent veins to ensure adequate blood supply to the flap and mitigate the risk of swelling by increasing backflow (28, 29). The proximal superficial vein served as the efferent vein, which promoted dermal backflow and minimized dermal swelling. The length of the afferent vein was minimized to avoid complications linked to venous valve damage. Moreover, the average thickness of modified iAVF was 2.3 mm (range: 1.6–3.0 mm) (Table 2), fulfilling the standard of superthin flaps without requiring additional thinning procedures. In conclusion, this method improved the survival rate and quality of iAVF, reduced the risk of complications, and represents a viable approach for the reconstruction of small- and medium-sized hand defects.

According to the concept of “replacement with like tissue” (30), the tissue used for hand reconstruction should be thin and wear-resistant. Therefore, for hand defects with exposed tendons, nerves, bones, or joints, skin grafts are not feasible. Pedicled island flaps, adipofascial flap, adjacent finger flaps, advancement flaps, and free flaps have inherent limitations, such as the need to sacrifice arterial supply, bulky postoperative flaps requiring secondary intervention, functional impairment secondary to scar formation, and poor aesthetic outcomes (1–5). Moreover, these flaps are also limited by flap size, rotation arc, defect location, number of damaged fingers, and pedicle length required for reconstruction (31). VF has been increasingly employed in traumatic hand defect reconstruction to address the aforementioned challenges (4, 7, 12, 23). Moreover, the modified iAVF eliminates the need for intricate perforator vessel dissection, making the flap readily accessible to surgeons who are not specialists in microvascular or micro-reconstructive surgery.

Compared with the uncertain survival rate reported in earlier studies, various methods (such as AVF, delayed surgery, pre-arterialization, and increasing the number of draining veins) significantly improved the survival rate of VF (13). Woo et al. (8) retrospectively analyzed 154 cases of hand defect reconstruction using AVF flaps and documented an AVF survival rate of 98.1%. Similarly, a systematic review involving 626 VFs described that the VF survival rate was approximately 95%, with 79.6% of flaps being complication-free (23). Of note, Wharton et al. (12) conducted a systematic review of 756 VFs and evinced that 86% of the surviving flaps had no complications. They also found that the survival rate of VF has improved since 2010, possibly owing to the increased experience of surgeons and further advancements in surgical techniques (12). It is worthwhile emphasizing that the current total necrosis rate of VF is 5.8% (23), comparable to the total necrosis rate of free arterial flaps of 4.4% (32). Taken together, these studies collectively demonstrate that the survival rate of VF, especially AVF, is comparable to that of arterial flaps, offering a safe and reliable alternative for hand defect reconstruction.

A systematic review undertaken by Wharton et al. (12) revealed that postoperative congestion and partial necrosis remain major complications in VF reconstruction. Indeed, the rate of early postoperative venous congestion was as high as 60%, whilst the partial necrosis rate was 10% (12). Likewise, Roberts et al. (23) uncovered that the postoperative partial necrosis rate of VF could reach 14.7%. Therefore, minimizing the risk of swelling and partial necrosis is an important research direction in the future. Koch et al. (14) employed retrograde AVF to repair 13 skin defects, and 6 flaps experienced venous congestion with epidermolysis. They indicated that venous communicating branches caused peripheral hypoperfusion of the VF, leading to a high risk of flap necrosis. This finding was also supported by indoline green angiography (6). However, retrograde AVF requires venous valve manipulation, thereby increasing surgical complexity and the risk of vascular damage (15–17). Moreover, Yu et al. (21) uncovered that retrograde iAVF did not lower the incidence of postoperative complications such as venous congestion and swelling. Therefore, retrograde AVF was not considered herein.

Lin et al. (18, 33) signaled that extensive direct arteriovenous communication branches elevate the risk of peripheral hypoperfusion of the VF and increase efferent venous pressure, promoting the occurrence of complications such as venous stasis, flap swelling, tension blister, unstable survival, and poor flap survival rates and quality. To limit the incidence of these complications, they developed a shunt-restricted AVF technique that blocks communicating veins (7, 18, 19, 33). Importantly, laser Doppler has validated that the shunt-restricted technique improves venous outflow, enhances flap perfusion, reduces the risk of complications, and prevents venous congestion (33). Herein, the shunt-restricted approach was further optimized, taking into account the deep and superficial two-layer system of the dorsalis pedis vein (34). Specifically, communicating branches between deep and superficial veins were blocked, which we hypothesize are the primary causes of dermal swelling and epidermolysis. Clinical results after blocking these communicating branches confirmed this hypothesis.

In theory, VF can be harvested from donor sites throughout the body. Nonetheless, donor sites reported in existing literature are limited to the inner forearm, inner calf, thenar region, back of hand, dorsum of foot, etc. (23). At present, the forearm is the most commonly used donor site, with a higher complete survival rate and a lower incidence of partial necrosis compared with other donor sites (23). However, compared with forearm AVF, iAVF offers several advantages for the reconstruction of soft tissue defects in hands, such as similar skin texture to the defect, minimal subcutaneous fat, easy-to-hide scars, ability to carry tendons and nerves, and larger incision are (11, 20–22). Moreover, the survival rate of VF is closely associated with a rich venous network (35–37), and the dorsum of the foot has an extensive venous network that facilitates efficient circulation. Zor et al. (22) described that the dorsal region of the foot possesses a rich venous network; consequently, they utilized iAVF to reconstruct 9 cases of palmar contracture. Despite the occurrence of partial necrosis in 2 cases and significant swelling in all flaps, their study demonstrated the viability of the dorsum of the foot as a VF donor site.

The shunt-restricted protocol was modified according to the anatomical characteristics of the dorsum of the foot vein to limit the risk of postoperative complications (Figure 1). Our case series demonstrated that modified iAVF used for hand defect reconstruction resulted in a high survival rate, low complication rate, and lower incidence of postoperative venous congestion. Although scar hyperplasia of the dorsal foot was evident in some patients, all patients expressed high satisfaction levels (Supplementary Table 1). Besides, the modified iAVF restored hand functionality after hand defect reconstruction. After an average follow-up period of 3.71 years, the MHQ scores were comparable between the injured hand and the healthy hand (Supplementary Table 1), reflecting favorable hand function recovery. Although the flap achieved reliable soft-tissue cover, donor-site morbidity should not be overlooked. In our case series, although FTSG harvest sites in some patients developed hypertrophic scarring, foot function remained completely unaffected. Future studies should compare donor-site outcomes across alternate reconstructive options (e.g., local fasciocutaneous flaps versus free flaps) to refine patient selection.

While the pathophysiology of venous flaps remains to be elucidated, there are three leading theories (13, 23): (1) arteriovenous shunting via dilated and deinnervated veins, (2) reverse flow through the capillary system, and (3) capillary bypass with rapid revascularization. Lam et al. (7) preliminarily explored the survival mechanism underlying shunt-restricted AVF and documented that at the proximal end of the restricted shunt, the high arterial pressure drives blood to flow backward into the capillaries and perfuse the periphery of the flap. However, at the distal end of the restricted shunt, low-pressure venous drainage promotes blood return through the capillaries to the venous system. Therefore, they proposed placing the restricted shunt at the proximal 1/3 of the flap (7, 19). We postulate that the blood flow pattern in modified iAVF is in line with the findings of Lam et al. (7) Following arterial anastomosis with the afferent vein, the deep vein was arterialized, and a portion of the blood flowed into the arterial system through the direct communicating branch between the artery and the vein, perfusing the flap in a physiological manner (38). Conversely, the remaining blood flowed retrograde through the venous network into the capillary network to perfuse the flap. Finally, the blood steadily flowed from the deep layer to the superficial layer and exited through the superficial veins, forming a “physiologically similar” perfusion pattern for the flap. Moreover, some non-functional small veins gradually dilate during the postoperative period, resulting in increased pressure in the efferent veins, which may have accounted for the mild congestion of the flap around the third day post-surgery.

Overall, our initial experience with 16 patients highlights the safety and feasibility of the modified iAVF for the reconstruction of hand defects. However, some limitations of this study merit acknowledgment. Firstly, the single-center design, small sample (n = 16) and the retrospective nature of the study, as well as the absence of a comparative control group may have compromised the findings of the present study. Consequently, any inferences regarding long-term safety and efficacy should be regarded as preliminary. Secondly, outcome assessors were not blinded to the surgical procedure, and all functional and aesthetic data were derived from self-reported MHQ scores. These factors may have introduced performance bias (care-giver) and reporting bias (patient). Blinded, clinician-graded measures were not collected, thereby limiting the objectivity of our findings. Finally, the survival mechanism of the modified iAVF remains hypothetical, given that it lacks objective validation. Nevertheless, modified iAVF represents a reliable reconstruction option for hand defects and offers significant therapeutic advantages. Further follow-up, multi- center and well-designed large-sample prospective studies are warranted to establish the indications of this technique and evaluate long-term outcomes. Moreover, future prospective studies should incorporate independent, blinded assessors and objective functional tests (e.g., dynamometry, range-of-motion goniometry) alongside patient-reported outcome measures to corroborate these preliminary findings.

Based on the uniquely deep and superficial venous systems of the dorsal foot and shunt-restricted modifications, a novel approach for iAVF was developed. This technique can significantly minimize the risk of complications such as venous congestion, swelling, and epidermolysis, as well as significantly improve the survival rate and reliability of AVF. Taken together, these results collectively demonstrate the safety and reliability of this strategy for hand defect reconstruction. The modified iAVF can meet the aesthetic and functional requirements of hand reconstruction.

## Data Availability

The original contributions presented in the study are included in the article/Supplementary material, further inquiries can be directed to the corresponding authors.
