# Supplementary material for: Long-term follow-up of modified shunt-restricted instep arterialized venous flap for reconstruction of hand defects
Source: Front Med (Lausanne). 2025 Aug 18;12:1662159. doi: 10.3389/fmed.2025.1662159 (PMC12399605; doi:10.3389/fmed.2025.1662159)
Supplement: Supplementary file 1 [file Table_1.docx]

Supplementary Table 1 Hand function assessment using MHQ

| No. | Total score | |  | OHF | |  | ALD | |  | Overall ADL | |  | WP | |  | Pain | |  | Aesthetics | |  | Satisfaction | |
| --- | --- | --- | --- | --- | --- | --- | --- | --- | --- | --- | --- | --- | --- | --- | --- | --- | --- | --- | --- | --- | --- | --- | --- |
|  | Injured | Healthy |  | Injured | Healthy |  | Injured | Healthy |  | Injured | Healthy |  | Injured | Healthy |  | Injured | Healthy |  | Injured | Healthy |  | Injured | Healthy |
| 1 | 100 | 100 |  | 100 | 100 |  | 100 | 100 |  | 100 | 100 |  | 100 | 100 |  | 0 | 0 |  | 100 | 100 |  | 100 | 100 |
| 2 | 100 | 100 |  | 100 | 100 |  | 100 | 100 |  | 100 | 100 |  | 100 | 100 |  | 0 | 0 |  | 100 | 100 |  | 100 | 100 |
| 3 | 100 | 100 |  | 100 | 100 |  | 100 | 100 |  | 100 | 100 |  | 100 | 100 |  | 0 | 0 |  | 100 | 100 |  | 100 | 100 |
| 4 | 98.19 | 100 |  | 100 | 100 |  | 95 | 100 |  | 97.5 | 100 |  | 100 | 100 |  | 0 | 0 |  | 100 | 100 |  | 100 | 100 |
| 5 | 100 | 100 |  | 100 | 100 |  | 100 | 100 |  | 100 | 100 |  | 100 | 100 |  | 0 | 0 |  | 100 | 100 |  | 100 | 100 |
| 6 | 99.4 | 100 |  | 100 | 100 |  | 100 | 100 |  | 100 | 100 |  | 96.43 | 100 |  | 0 | 0 |  | 100 | 100 |  | 100 | 100 |
| 7 | 93.22 | 100 |  | 95 | 100 |  | 95 | 100 |  | 93.93 | 100 |  | 95 | 100 |  | 10 | 0 |  | 93.75 | 100 |  | 91.67 | 100 |
| 8 | 98.96 | 100 |  | 100 | 100 |  | 100 | 100 |  | 100 | 100 |  | 100 | 100 |  | 0 | 0 |  | 100 | 100 |  | 100 | 100 |
| 9 | 100 | 100 |  | 100 | 100 |  | 100 | 100 |  | 100 | 100 |  | 100 | 100 |  | 0 | 0 |  | 100 | 100 |  | 100 | 100 |
| 10 | 100 | 100 |  | 100 | 100 |  | 100 | 100 |  | 100 | 100 |  | 100 | 100 |  | 0 | 0 |  | 100 | 100 |  | 100 | 100 |
| 11 | 100 | 100 |  | 100 | 100 |  | 100 | 100 |  | 100 | 100 |  | 100 | 100 |  | 0 | 0 |  | 100 | 100 |  | 100 | 100 |
| 12 | 100 | 100 |  | 100 | 100 |  | 100 | 100 |  | 100 | 100 |  | 100 | 100 |  | 0 | 0 |  | 100 | 100 |  | 100 | 100 |
| 13 | 100 | 100 |  | 100 | 100 |  | 100 | 100 |  | 100 | 100 |  | 100 | 100 |  | 0 | 0 |  | 100 | 100 |  | 100 | 100 |
| 14 | 100 | 100 |  | 100 | 100 |  | 100 | 100 |  | 100 | 100 |  | 100 | 100 |  | 0 | 0 |  | 100 | 100 |  | 100 | 100 |
| 15 | 100 | 100 |  | 100 | 100 |  | 100 | 100 |  | 100 | 100 |  | 100 | 100 |  | 0 | 0 |  | 100 | 100 |  | 100 | 100 |
| 16 | 100 | 100 |  | 100 | 100 |  | 100 | 100 |  | 100 | 100 |  | 100 | 100 |  | 0 | 0 |  | 100 | 100 |  | 100 | 100 |
| Median (range) | 100  (93.22, 100) | 100 |  | 100  (95.00, 100) | 100 |  | 100 95.00, 100) | 100 |  | 100 93.93, 100) | 100 |  | 100 (95.00, 100) | 100 |  | 0 (0-10) | 0 |  | 100 (93.75,100) | 100 |  | 100 (91.67, 100) | 100 |
| *P*-value  MHQ: the Michigan Hand Questionnaire. OHF: overall hand function; ADL: activities of daily living; WP: work performance. The *p* value was obtained by comparing the MHQ score of the injured side with the score of the healthy side after follow up. *Statistically significant, *p* < 0.05. | 0.068 | |  | 0.317 | |  | 0.157 |  |  | 0.180 | |  | 0.180 | |  | 0.317 | |  | 0.317 | |  | 0.317 | |
